# Enhanced Expression of Full-Length Human Cytomegalovirus Fusion Protein in Non-Swelling Baculovirus-Infected Cells with a Minimal Fed-Batch Strategy

**DOI:** 10.1371/journal.pone.0090753

**Published:** 2014-03-04

**Authors:** Marco Patrone, Nuno Carinhas, Marcos Q. Sousa, Cristina Peixoto, Claudio Ciferri, Andrea Carfì, Paula M. Alves

**Affiliations:** 1 iBET, Instituto de Biologia Experimental e Tecnológica, Oeiras, Portugal; 2 Instituto de Tecnologia Química e Biológica, Universidade Nova de Lisboa, Oeiras, Portugal; 3 Novartis Vaccines and Diagnostics, Cambridge, Massachusetts, United States of America; University of Regensburg, Germany

## Abstract

Human cytomegalovirus congenital infection represents an unmet medical issue and attempts are ongoing to develop an effective vaccine. The virion fusion players of this enveloped virus are the natural targets to achieve this goal and to develop novel anti-viral therapies. The secreted ectodomain of the viral fusion factor glycoprotein B (gB) has been exploited so far as an alternative to the cumbersome expression of the wild type trans-membrane protein. In the soluble form, gB showed encouraging but limited potential as antigen candidate calling for further efforts. Here, the exhaustive evaluation of the Baculovirus/insect cell expression system has been coupled to an orthogonal screening for expression additives to produce full-length gB. In detail, rapamycin was found to prolong gB intracellular accumulation while inhibiting the infection-induced cell swelling. Not obvious to predict, this inhibition did not affect Baculovirus growth, revealing that the virus-induced cell size increase is a dispensable side phenotype. In parallel, a feeding strategy for the limiting nutrient cysteine has been set up which improved gB stability. This multi-modal scheme allowed the production of full-length, mutation-free gB in the milligram scale. The recombinant full-length gB obtained was embedded into a stable mono-dispersed particle substantially larger than the protein trimer itself, according to the reported association of this protein with detergent-resistant lipid domains.

## Introduction

The pathogenic role of Human cytomegalovirus (HCMV) in a wide range of human diseases has raised several concerns over the last years. Indeed, HCMV infection is a major cause of morbidity and mortality in humans with acquired or developmental deficits in innate and adaptive immunity [1]. Moreover, the social and economic costs due to the low efficacy of the available therapies are becoming evident. Consequently, this herpesvirus is among the top three infectious human pathogens for which the development of a vaccine is stated as a priority [2], [3].

Enveloped virions, like those produced by herpesvirus-infected cells, start a new infection cycle by performing the host cell receptor-dependent merging of the virion envelope with a target cellular membrane [4]. Hence, the trans-membrane viral fusion mediators are targets for vaccine development and it became recently clear that glycoprotein B (gB) is the protein carrying the membrane merging activity in herpesviruses [5]. After many years of unsuccessful attempts, an adjuvated CMV vaccine candidate based on a secreted form of gB has been reported to reduce by 50% the infection risk in a Phase II clinical trial [6]. Prompted by this important achievement, a renovated effort is ongoing worldwide seeking for strategies to increase the efficacy of CMV vaccine candidates beyond the threshold for cost-effectiveness, estimated around the 60% coverage [7].

HCMV gB undergoes an extensive rearrangement during the virion-mediated membrane merging [8], as a consequence of a still undefined triggering interplay with the ancillary gH/gL/gpUL131A/gpUL130/pUL128 receptor-binding complex [8]–[11]. In this respect, the evidence that secreted gB ectodomain (gB_ecto_) adopts a trimeric post-fusion conformation [12] suggests that gB_ecto_ might miss strong conformation-dependent neutralization epitopes [13]. The latter hypothesis is further supported by the direct role of the C-terminal endodomain in regulating gB-mediated membrane fusion [14], [15].

HCMV gB, like all gB homologs from human herpesviruses except HSV-1 and -2, is synthesized as a precursor polypeptide cleaved by furin into the N-terminal surface (SU) and the C-terminal membrane-anchored (TM) chains, which are held together by one out of the five disulfide bridges present [12], [16] and anchored to membranes by the single-pass hydrophobic helix of the TM chain. Furthermore, HCMV gB is heavily glycosylated with 18 potential N-glycosites and ca. 40% of its mass is composed by sugars [17], when expressed in human cells. The sum of the several post-translational modifications HCMV gB undergoes, along with its overall large molecular size (360–420 kDa *per* trimer, depending on the expression host), has hampered so far the quantitative production of full-length gB in recombinant form (hereafter FL-gB), thus slowing its detailed and broad understanding for a full biomedical exploitation of this essential target.

The Baculovirus Expression Vector System (BEVS) has been extensively used for biochemical and structural studies of many difficult targets (see [18] and references therein). The reduced cost as compared to mammalian expression systems has made the insect cell platform a common choice for vaccine development. In particular, BEVS has been used for two of the most recently approved recombinant vaccines, Cervarix® and FluBlok® [19], [20]. Traditionally, the secreted hydrophilic ectodomains derived from the herpesviral fusion players have been considered easier to produce than their full-length counterparts [4]. Nevertheless, the expression of EBV and HCMV gB_ecto_ proteins required the mutagenesis of their respective fusion loops to avoid aggregation and to achieve satisfactory yields and quality [21], [22]. While the resulting engineered proteins have been of paramount importance to build up a unifying picture of the post-active conformation in herpesviral gB homologs and class III fusion proteins [23], they lack all the pivotal lipid-interacting and modulatory elements of these viral factors.

Here, we show that, by the systematic investigation of the highly flexible multi-parametric space offered by BEVS – coupled with a mild and easy-to-run downstream design – a stable and essentially mono-dispersed, mutation-free HCMV FL-gB was obtained. Prominently, we also report that the TOR inhibitor rapamycin increased FL-gB cell-specific expression and prevented the cytopathic effect induced by Baculovirus, without interfering with the infection kinetics. Moreover, supplementing the culture medium with additional cysteine further improved FL-gB production. Worth to note, the volumetric productivity achieved with the above strategy (0.35 mg/L) is very similar to that reported for the multi-mutated HCMV gB_ecto_ deletion variant [22]. Finally, we proved that the developed procedure is scalable and it can be transferred to pilot bioreactors.

## Materials and Methods

### Cell Cultures and Recombinant Baculovirus

Suspension cultures of *S. frugiperda* Sf9 (ECACC 89070101) and *T. ni* BTI-TN5B1-4 (High Five™, Life Technologies, hereafter abbreviated High Five) cell lines were maintained in SF900II (Life Technologies) or Insect-XPRESS™ (Lonza) serum-free media, respectively, and grown at 27°C in orbital shaking incubators. Cell counts and viability were routinely measured by Trypan Blue exclusion in a Fuchs-Rosenthal chamber (Brandt). Cells were sub-cultured every 3–4 days by diluting ≥95% viable cultures at 0.5×10^6^ cell/mL for Sf9 or at 0.3×10^6^ cell/mL for High Five.

For bioreactor runs, both cell lines were inoculated at 0.5×10^6^ cell/mL and dissolved oxygen (DO) set at 30% of air saturation. Temperature was 27°C for Sf9 or 29°C for High Five. Cells were grown up to the desired cell concentration at the time of infection (CCI). Water jacket stirred tank bioreactors BIOSTAT® QPlus (for 0.5 L culture volume) and BIOSTAT® B-DCU (for 1 L culture volume, both from Sartorius AG) were operated with a stirring range of 90–180 rpm for Sf9 or 90–270 rpm for High Five; DO was controlled by sequential N_2_-stirring-O_2_ cascade mode with 0.01 vessel volume·min^−1^ air-flow rate. Rocking WAVE 20/50EHT Bioreactor™ (50 L bag for 25 L culture volume, GE Healthcare) was used for up-scaling FL-gB production in High Five and operated via UBICON at 10 to 22 rpm with 7° rocking angle and 0.1 to 0.3 Liter·min^−1^ aeration rate; 300x concentrated cysteine feed was prepared in culture medium and provided in a continuous fashion through a gravimetric controlled pump at 100 mL·day^−1^ starting at 24 hours after the infection.

Primers listed in Table 1 have been used to amplify the HCMV UL55 ORF from FIX7 BAC [24], replacing the wt stop codon with an in-frame 93 bp-long sequence coding for a C-terminal Twin-Strep-tag® by 5′ extension PCR with *Pfu* DNApol (Promega). The DNA sequence coding for the tag was manually adapted based on Gene Designer 2.0 software (DNA 2.0 Inc.).

**Table 1:** Primers used to construct bBst2x^1^.

Fw	5′-GGCGC**GGATCC**atgGAATCCAGGATCTGGTGCCT-3′
Rv 1	5′-TTGCGGGTGGCTCCAGGCCGAGACGTTCTCTTCTTCGTCAGAGTC-3′
Rv 2	5′-*CCCGAGCCACCGCCTTTTTCAAA* TTGCGGGTGGCTCCAGGCCGA-3′
Rv 3	5′-GCAGAGCCTCCCGAGCCTCCA *CCCGAGCCACCGCCTTTTTCAAA*-3′
Rv 4	5′-*TTTTCGAATTGCGGATGCGACCAC* GCAGAGCCTCCCGAGCCTCCA-3′
Rv 5	5′-CCC**AAGCtt**aC*TTTTCGAATTGCGGATGCGAC*-3′

1 Fw and Rv stand for forward and reverse primers, respectively; restriction sites are in boldface; start and stop codons are in lowercase; overlapping sequences for 5′ extension PCR are matched as underlined and italics.

After sequence validation of the resulting UL55strep2x, recombinant *Ac*MNPV expressing Strep-tagged FL-gB under *polh* promoter (termed bBst2x) was generated by Tn7-mediated transposition according to Bac-to-Bac® system guidelines (Life Technologies). Infectious bBst2x was reconstituted by transfecting adherent Sf9 cells and the supernatant collected after 48 hours (generation V0). Viral stocks were amplified and expanded until generation V2 by infecting suspension Sf9 cell cultures at CCI 0.5 with 0.1 multiplicity of infection (m.o.i.). Viral progenies were titrated by plaque assay.

### Analyticals for Upstream Process Development

Cell volume was measured with Casy® TTC (Schärfer). Glucose, glutamine, lactate and glutamate concentrations in cell cultures were monitored off-line by YSI 7100 MBS (Yellow Springs Instruments).

FL-gB and *Ac*MNPV gp64 cell number-specific expression kinetics were followed by immunoblot. Unless otherwise stated, clarified detergent protein extracts from 10^5^ cell equivalents were loaded onto 4–12% Novex® NuPAGE® Bis-Tris pre-cast gels (Life Technologies) followed by blotting onto a nitrocellulose membrane. Mouse monoclonal clone 27–78 against HCMV gB TM chain was a generous gift from W. Britt (University of Alabama, Birmingham AL). Mouse monoclonal anti-*Ac*MNPV gp64 clone AcV5 was from eBioscience. Chemiluminescent signals were recorded at ChemiDoc XRS+ (Bio-Rad). Relative image analysis of FL-gB precursor and TM chain band intensities from the same immunoblots was by Quantity One® 1-D software (Bio-Rad). To compensate for inconsistent transfer efficiency of the gB uncleaved form and chemiluminescent development between different blots, samples from different experimental series were loaded in the same gel to normalize for their signal intensities.

### Downstream Purification and Analysis of FL-gB

FL-gB was extracted from the microsome fraction of bBst2x-infected cells in 100 mM Tris·HCl, 0.15 mM NaCl, 0.05% w/v cymal-5 (Anatrace Affymetrics), 20% glycerol, 2.5 mM dithiothreitol, pH 8 (FL-gB buffer) supplemented with 1% w/v dodecyl maltoside, 1.25 U/mL Benzonase® (Merck Millipore), 20 µg/mL avidin and cOmplete protease inhibitor cocktail (Roche). Insoluble material was sedimented by 100,000×*g* ultracentrifugation for 1 h at 4°C. The clarified extract was concentrated four fold in a 100 k NMWCO VivaFlow cassette (Sartorius AG) before being loaded onto a *Strep*Tactin™ column (GE Healthcare) at 12 cm/h flow velocity. The resin was then washed until UV baseline in FL-gB buffer and the protein eluted with 2.5 mM desthiobiotin. Pooled positive fractions were injected in a 16/600 Superdex 200 column (GE Healthcare) at 1 mL/min of FL-gB buffer. The column for size exclusion chromatography was calibrated with bovine serum albumin, alcohol dehydrogenase, ferritin, thyroglobulin, blue dextran 2000 (Gel Filtration HMW Calibration kit, GE Healthcare) dissolved in FL-gB buffer. Final yields were 9 mg of FL-gB at 4 mg/mL from 25 L culture, with a volumetric productivity of 0.35 mg/L.

Mass spectrometry was performed at the UniMS Facility of iBET/ITQB. Briefly, gel bands were in-gel tryptic digested and the extracted peptides were loaded onto a R2 micro column and eluted directly onto a MALDI plate using α-ciano-4-hydroxycinamic acid in 50% acetonitrile and 5% formic acid. MS and MS/MS modes were run on a 4800 Plus MALDI TOF/TOF™ Analyzer (AB SCIEX). The collected MS and MS/MS spectra were filtered for trypsin autolysis peaks and assigned with Mascot software (Matrix Science) against NCBI database (50 ppm peptide mass tolerance) without taxonomy restrictions.

Furin cleavage was carried out by incubating 5 µg of FL-gB at 30°C for 4 h with the indicated units of recombinant furin enzyme (New England Biolabs).

Deglycosylation assay was either with PNGase F (Roche) after FL-gB denaturation or with endoglycosidases F1 F2 F3 (ENDEGLY, Sigma) in native conditions, according to the respective manufacturers’ guidelines.

Blue native protein electrophoresis (BN-PAGE) was performed by loading 10 µg of FL-gB in 4–16% NativePAGE™ Novex® Bis-Tris pre-cast gel system (Life Technologies).

#### Electron microscopy analysis

4 µl of purified sample was placed onto a continuous carbon grid previously glow discharged (EMS, Hatfield, PA USA). After 30 s of incubation, the sample was negatively-stained with a solution of 2% uranyl formate and blotted dry. Samples were imaged using a Tecnai T12 Spirit operating at 120 keV at a nominal magnification of 49,000× (3.14 Å/pixel at the detector level) using a defocus range of −0.6 to −1.3 µm. Images were recorded on a Gatan 4096×4096 pixel CCD camera (15 µm pixel size). Particles were isolated using the EMAN2 e2boxer algorithm (Tang et al., 2007) and extracted with a box size of 224×224-pixels. Iterative multivariate statistical analysis (MSA) and multi-reference alignment (MRA) of the extracted particles provided representative 2D views of the FL-gB.

## Results

### Preliminary Screening for FL-gB Expression Conditions

**Figure 1:**
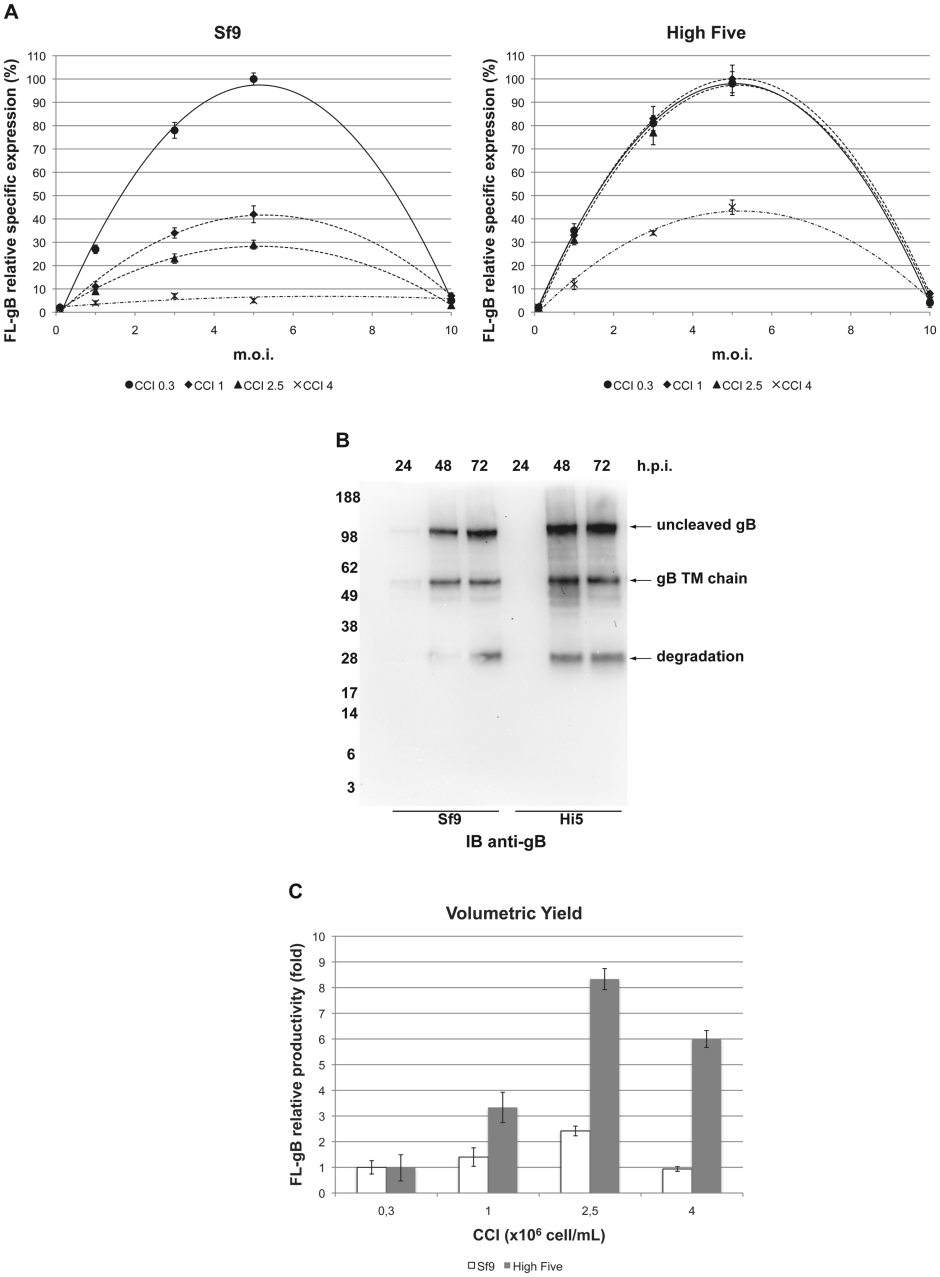
BEVS-driven FL-gB expression screening. (**A**) bBst2x was used to infect Sf9 (left panel) or High Five (right panel) cells at 0.3 (circles), 1 (diamonds), 2.5 (triangles) or 4 (crosses) ×10^6^ cell/mL (CCI)) with mutliplicities of infection (m.o.i.) of 0.1, 1, 3, 5 or 10 pfu/cell. Cells were harvested at 72 hours after infection and detergent-soluble protein extracts were analysed by densitometric analysis of anti-gB TM immunoblots. Overall relative quantification was made by comparing samples from different experimental series in the same immunoblots (hereafter n = 3 with error bars representing 95% confidence interval, C.I.) and setting as 100% the mean of most intense signals. (**B**) Comparison of FL-gB expression pattern and kinetics in Sf9 and High Five cell lines, bBst2x-infected with m.o.i. 5 at CCI 0.3 (Sf9) or 2.5 (High Five, Hi5). Cells were sampled at the indicated hours *post*-infection (h.p.i.) and detergent-soluble cell extracts probed in immunoblot (IB) with anti-gB TM. Positions of the molecular weight markers are indicated and expressed in kDa. (**C**) Theroretical FL-gB relative volumetric yields computed from (A) for m.o.i. 5 series, according to CCI and cell viability at 72 h.p.i.

Thorough evaluation of the core set of variables provided by viral-vectored expression systems in the two insect cell lines most frequently used with BEVS showed that FL-gB levels increased up to saturating viral load in both Sf9 and High Five cells (Fig. 1A). Before turning-down at very high infectious doses (m.o.i. 10), FL-gB specific expression at 72 hours *post*-infection (h.p.i.) was proportional to the applied m.o.i., thus ruling out issues related to negative feed-back by protein over-expression or to transgene instability. On the other hand, Sf9 cells displayed a severe density effect, being unable to keep up FL-gB specific expression when raising the CCI. In contrast, High Five cells could maintain similar levels when infected at up to 50% of their cell concentration peak in growing cultures (routinely about 4.5–5×10^6^ cell/mL). This comparison suggests that FL-gB biosynthesis demanded a very active metabolic state of the host cell and confirms in this respect that High Five are better recombinant protein producers than Sf9 upon Baculovirus infection.

The improved performance of *T. ni* derived cells did not correlate either with a better expression kinetics nor with any general difference in FL-gB *post*-translational modifications or intracellular stability (Fig. 1B). When compared for similar expression levels, in both cell lines FL-gB accumulation reached a steady state at as soon as 48 hours *post*-infection without further increase at a later time point. The electrophoretic pattern was also equivalent showing incomplete proteolytic maturation of the 120 kDa precursor into the 55 kDa TM chain (SU chain is not detected by immunoblot with the monoclonal antibody used here) along with a 30 kDa degradation fragment. Therefore, the productivity was not a function of the expression quality which appeared an invariant feature of gB in this expression system.

Taken together, the above described evaluation identified the most favourable intersection of the basic parameters (Fig. 1C). The theoretical volumetric productivities were compared as computed based on CCI, cell viability and relative FL-gB levels and the setting giving the highest value (High Five, CCI 2.5 with m.o.i. 5) considered as the starting point to look for additional enhancement.

### Rapamycin Extends the Time Window of FL-gB Accumulation and Inhibits Infection-induced Cell Swelling

Since the discovery of its target, rapamycin has been widely exploited to investigate the several eukaryotic cellular pathways the highly conserved Ser/Thr kinase TOR links to cell metabolism and survival (see for instance [25], [26] and cited references). This bacterial toxin has also been used as an additive to improve viability and performance of *in vitro* cell cultures, including protein expression [27].

**Figure 2:**
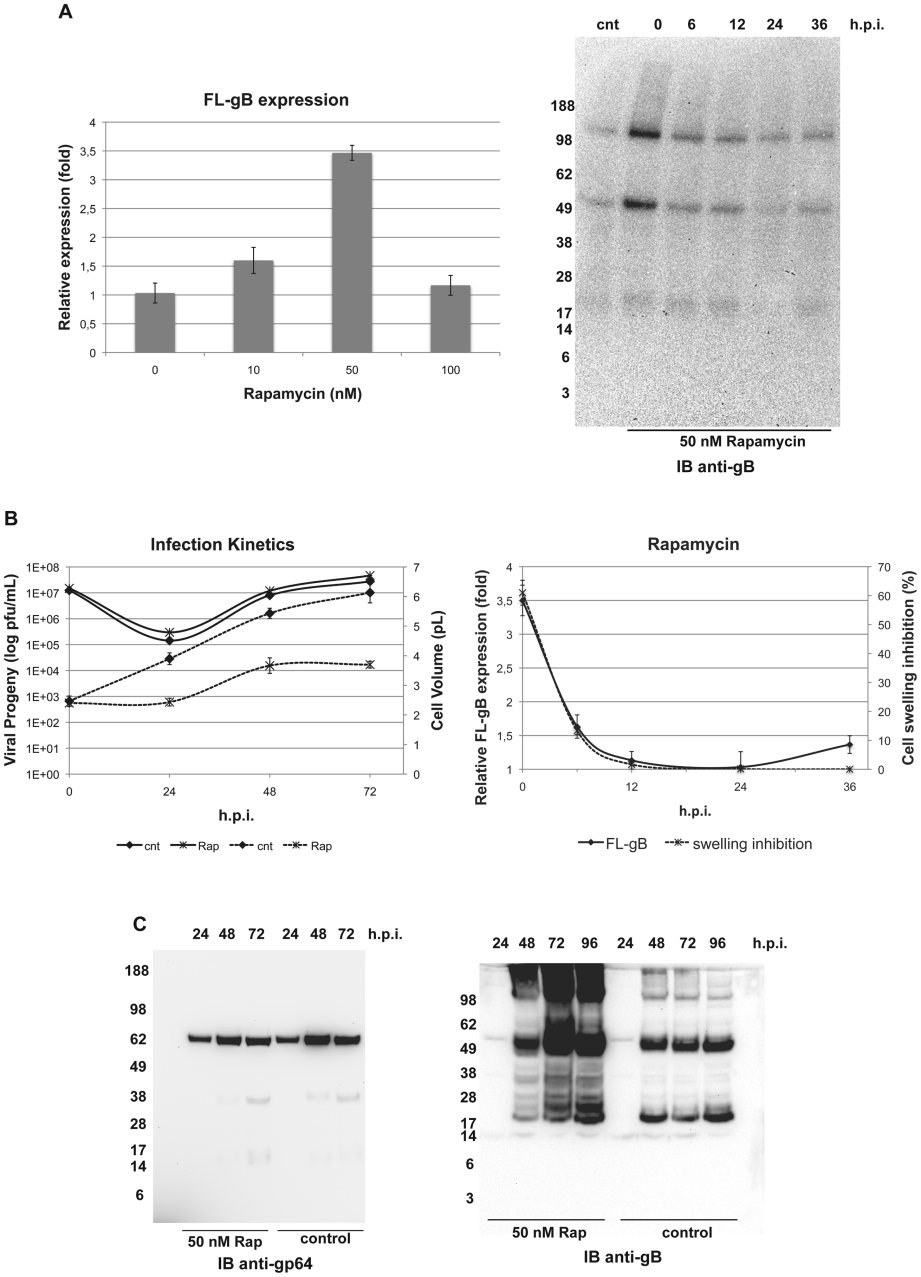
Effect of rapamycin on FL-gB expression. (**A** left panel) High Five cells were infected with bBst2x at CCI 2.5 with m.o.i. 5 in the presence of either 0.1% DMSO (cnt) or the indicated final concentrations of rapamycin, harvested at 72 h.p.i. and relative FL-gB expression analysed by immunoblot with ECL mean signals of control samples set as 1; (right panel) anti-gB TM immunoblot on detergent-soluble protein extracts from High Five cells infected as above in the presence of 0.1% DMSO (cnt) or with 50 nM rapamycin added at the indicated time points. (**B** left panel) High Five cells were infected as in (A) in the presence of 50 nM rapamycin (diamonds) or DMSO alone (stars) and cell cultures were sampled at the indicated h.p.i. to measure viral titers (solid lines) and cell volume (dashed lines); (right panel) High Five cell were infected as in (A right panel) and the recorded cell volume plotted as cell swelling inhibition (stars) along with relative FL-gB expression (diamonds) detected in the same culture samples. (**C**) Kinetic expression of FL-gB and *Ac*MNPV gp64 analysed by immunoblot of detergent-soluble protein extracts from High Five cell cultures infected and sampled as in (B left panel).

Rapamycin was found to increase FL-gB content in bBst2x-infected High Five cells at 72 h.p.i. with respect to the control (Fig. 2A, left panel). When added at moderate, subtoxic concentrations (50 nM) at the time of infection with m.o.i. 5 and CCI 2.5, the presence of rapamycin resulted in a significant enhancement of gB expression. This effect was strictly dependent on the time of rapamycin addition with respect to the infection stage (Fig. 2A, right panel). Indeed, rapamycin-induced gB boost was detected only when added at the time of infection. Adding the compound from 6 h.p.i. onwards did not lead to any major influence on the recombinant protein accumulation.

When analysed for a phenotypic outcome on the Baculovirus infection (Fig. 2b, left panel), the viral growth curve obtained from cells infected in the presence of 50 nM rapamycin overlapped that from the DMSO vector control. Consequently, gB-expression enhancing ability of rapamycin was not due to an interference with Baculovirus infectivity or virion assembly/release. In contrast, rapamycin-treated infected cells displayed a reduced cell size with respect to the control. Infection-induced cell swelling is a well known cyto-morphological alteration caused by many viruses, including *Ac*MNPV. Actually, the cytophatic effect is often used to empirically assess for effective viral growth. In control infections, the cell volume swelled more than twice, changing with a linear progression from 2.5±0.15 pL at 0 h.p.i. up to 6.1±0.3 pL 72 hours after the infection. In the presence of rapamycin, cell volume increased by 54% only at the end of the time course (3.7±0.1 pL) and the swelling was inhibited by ≥60% with respect to the control.

Interestingly, both gB expression enhancement and cell swelling inhibition were similarly dependent on the time of rapamycin addition (Fig. 2B, right panel). A remarkable correlation between the two responses elicited by this TOR inhibitor was found, with a negligible effect when added after the immediate early infection phase. Cell growth is among the processes influenced by the TOR-mediated axis and the loss of ability to reduce the infection-dependent cytopathy suggests that already infected cells become unresponsive to rapamycin, which coherently fails to increase FL-gB expression.

As suggested by the viral growth curves, rapamycin did not change the expression profile of the late gene-encoded *Ac*MNPV gp64 envelope protein, whereas FL-gB displayed an extended accumulation kinetics (Fig. 2C) peaking at 72 hours *post*-infection. This bell-shaped trend –indeed typical for transient over-expression systems– induced by rapamycin was in contrast to the flat gB profile obtained in the control. The latter evidence details the observed positive effect that rapamycin addition had on FL-gB intracellular levels in bBst2x-infected High Five cells.

### Cysteine Supplementation Further Improves gB Yields by Increasing the Detergent-soluble Protein Fraction

A number of nutrients were shown to be limiting for *in vitro* insect cell cultures used with BEVS [28]. In particular, cystine depletion was proposed to play a critical role in both insect cell proliferation and Baculovirus-driven recombinant protein expression.

Cystine is the form in which cysteine (Cys) is mainly provided to cell cultures due to the oxidation of this amino acid when exposed to the air. Nevertheless, the low solubility of cystine at physiologic pH values (0.5 mM) prevents its use at higher concentrations in culture media formulations, although it is well known that Cys is the limiting substrate for glutathione biosynthesis.

**Figure 3:**
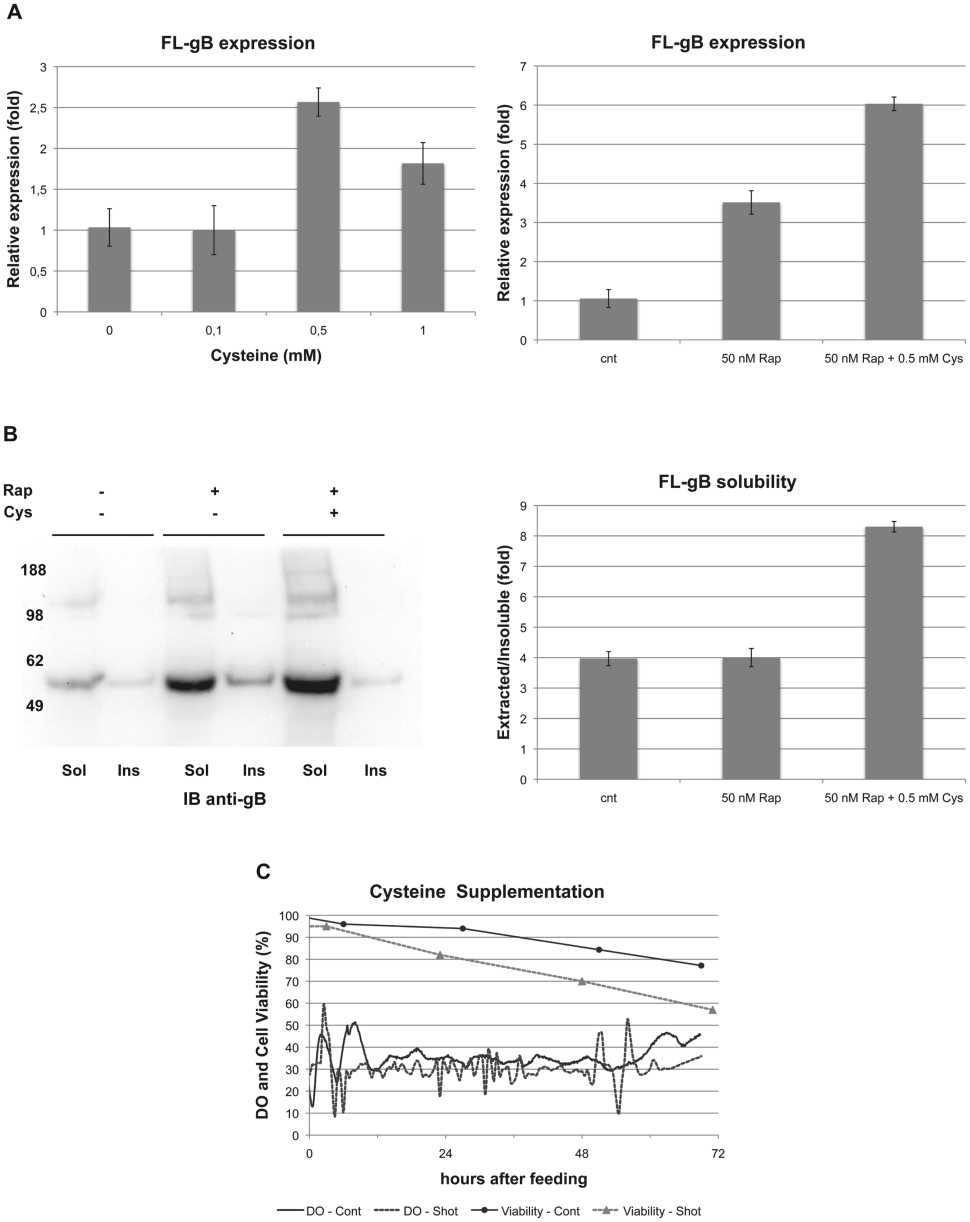
Cysteine supplementation further increases FL-gB expression in infected High Five. (**A**) 300x concentrated cysteine supplement was prepared in culture medium to obtain the indicated final Cys concentrations and added alone to High Five cell cultures 24 h.p.i. with bBst2x at CCI 2.5 with m.o.i. 5. Cys was added alone (left panel) or in combination with 50 nM rapamycin treatment (right panel). Cells were harvested at 72 h.p.i. and detergent-soluble FL-gB relative quantification performed as already described (controls were made by adding fresh medium to infected cell cultures previously supplemented or not with 0.1% DMSO at the time of infection). (**B**) FL-gB expression was compared in detergent-soluble (Sol) or -insoluble (Ins) protein extracts obtained from cells treated as in (A right panel). Equal loading was by resuspending the insoluble pellets in the same volume used to prepare the soluble fractions. Immunoblot (left panel) and densitometric analysis (right panel) are shown. (**C**) Effect on the dissolved oxygen (DO) control and cell viability in bioreactors of bBst2x-infected High Five - CCI 2.5, m.o.i. 5, rapamycin 50 nM - by either cysteine shot addition (Shot, dashed lines and triangles) or continuous feeding (Cont, solid lines and circles). Bioreactors were sampled for cell viability, while DO was recorded in real-time and expressed as the percentage of air saturation.

Supplementing the culture medium with additional 0.5 mM Cys (final concentration) led to an increase of FL-gB expression over the basal level assessed 72 hours after infecting High Five cells with bBst2x (Fig. 3A, left panel). Furthermore, Cys supplementation was compatible with the boost induced by rapamycin (Fig. 3A, right panel), resulting in an additive effect on FL-gB expression when both strategies were combined.

A minor fraction of FL-gB expressed in the control was insoluble after the detergent extraction of the cellular proteins (Fig. 3B). Rapamycin enhanced the overall amount of gB expressed but did not change the extracted to insoluble protein ratio. On the other hand, supplementing with Cys increased detergent-solubilised gB relative to the fraction that remained non-extracted, the latter likely due to misfolding. The increment in the FL-gB detergent-soluble/insoluble ratio accounted for the additive action of Cys supplementation on the rapamycin-boosted gB productivity (compare left panels from Fig. 3A and 3B, respectively), thus suggesting that the benefit was originated from a higher folding efficiency and/or fold stability of FL-gB.

The scale-up of the developed strategy pointed out that cysteine bolus addition to the infected cell culture hindered a proper oxygen control in bioreactors (Fig. 3C). All-in-one supplementation with 0.5 mM Cys triggered the DO controller into an offset loop that could not be compensated and ultimately resulted into poor cell viability at the end of the process (<60%). To overcome the interference Cys oxidation caused to DO control, a continuous fed-batch was set up to provide about 170 µmol Cys ·L^−1^·day^−1^ over 72 hours (Cys concentrated feed was stable as Cystine precipitate was not observed). After an initial adjustment phase, the constant feeding strategy allowed to maintain DO closer to the set point (30% of air saturation) with smoother oscillations and achieving a cell viability slightly lower than 80% at the time of harvesting (TOH).

### Purified FL-gB Arranges into a Large Stable Particle

The upstream procedure developed above was implemented in a 25 L pilot scale bioreactor and 9 mg of FL-gB were purified (see Material and Methods) and analysed.

**Figure 4:**
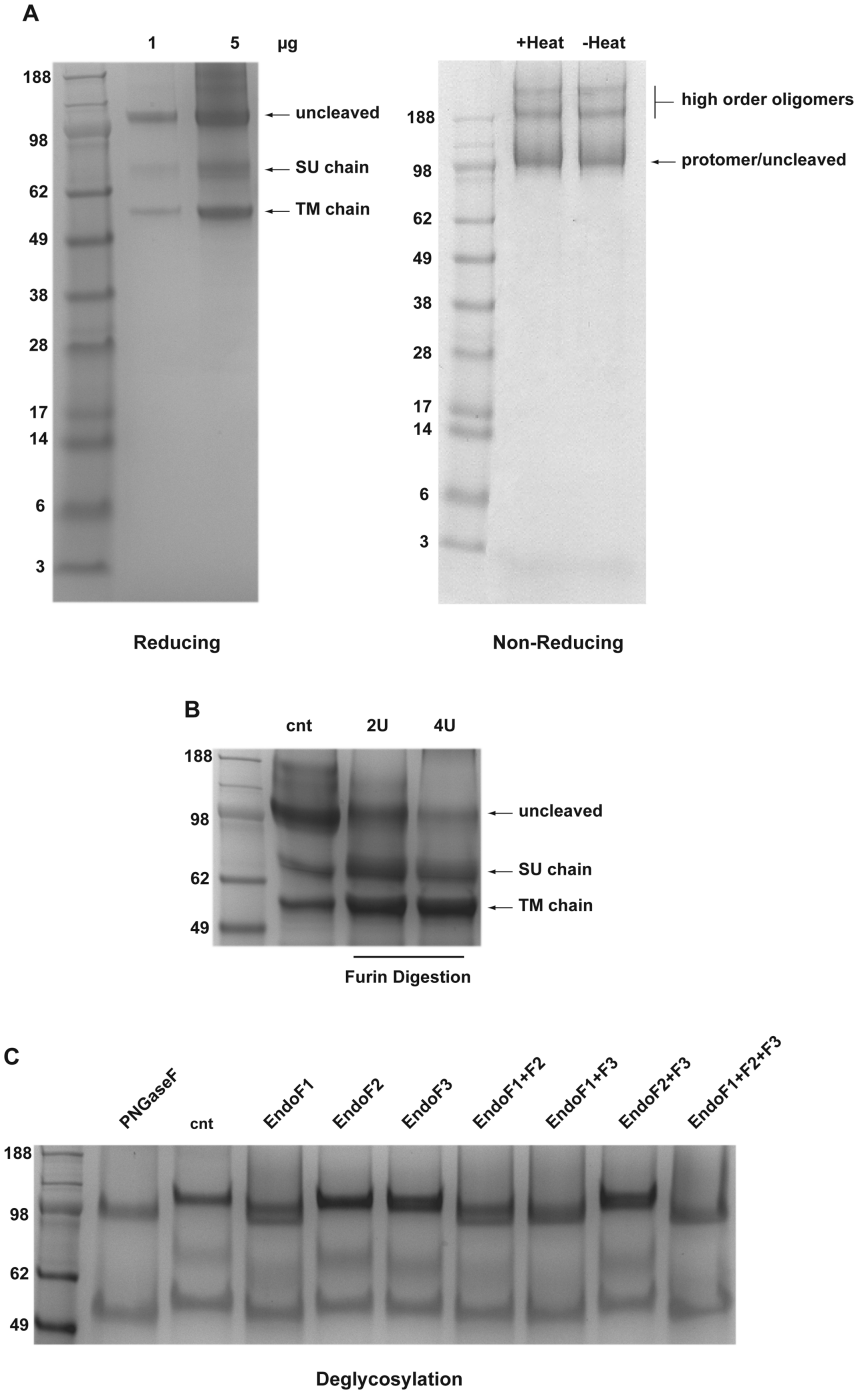
Analysis of purified FL-gB. (**A**) Purified reduced (left panel) or non-reduced (right panel) FL-gB was loaded on SDS-PAGE; amounts of FL-gB loaded in the reducing SDS-PAGE are indicated; for non-reducing electrophoresis, 2.5 µg of FL-gB were heated or not in the absence of reducing agents. The positions of the uncleaved FL-gB, TM and SU chains are indicated as migrating before or after the reduction of the gB protomer disulfide bridges. (**B**) 5 µg of purified FL-gB were analysed by reducing SDS-PAGE after incubating the protein for 4 hours at 30°C with or without the indicated units (U) of recombinant furin and loaded on reducing SDS-PAGE. (**C**) FL-gB glycosylation was analysed by incubating 2.5 µg of either denatured or native FL-gB with PNGase F for 3 hours or endoglycosidase F isoforms for 1 hour, respectively; the control sample was left undigested. Denaturation was by heating FL-gB at 99°C for 20 min in 0.2% SDS and 1% 2-mercaptoethanol.

Three bands of 120, 65 and 55 kDa were visible in reducing SDS-PAGE (Fig. 4A, left panel). The identity of the three polypeptides was assigned by mass spectrometry performed on the gel-extracted protein bands (Table 2) as the FL-gB uncleaved full length (120 kDa), the SU chain (65 kDa) and the TM chain (55 kDa). The proteolitically processed SU-TM FL-gB protomer co-migrated with the uncleaved form in non-reducing electrophoresis, thus showing the homogeneity of the two gB populations but the cleavage. Notably, the prominent heat-labile non covalent oligomeric form described for the secreted HCMV gB_ecto_ variant [22] was not visible in FL-gB (Fig. 4A, right panel), suggesting that the central coiled-coil featuring the post-fusion gB ectodomain [12], [21] was not present in the obtained full-length protein. Instead, high order covalent oligomeric forms were present in low abundance, rather resembling the pattern observed for gB extracted from the HCMV virion [29].

**Table 2:** gB peptides identified by MS/MS.

Sample	gB peptides identified (aa)^1^	C.I. %^2^
120 kDa band	93–107	100
	117–137	100
	152–181	99.958
	476–490	100
	496–509	98.506
	822–852	99.78
65 kDa band	152–181	99.942
	192–203	99.849
55 kDa band	476–490	100
	496–509	99.996
	521–538	99.966
	672–683	99.874
	803–821	100
	822–852	100

1 start and end amino acids as by conceptual translation of HCMV UL55 ORF.

2 Confidence Interval.

The uncleaved precursor present in the purified FL-gB preparation was quantitatively processed into additional amounts of the 65 and 55 kDa protein bands by recombinant furin (Fig. 4B), thus confirming the MS data. We also incidentally observed that, for an unknown reason, the Ca^2+^ present in the furin digestion buffer (1 mM) sharpened the electrophoretic mobility of both uncleaved FL-gB and SU chain, otherwise migrating as slightly broad bands. The smeared appearance of the two forms was then not due to heterogeneous occupancy of the N-glycosylation sites or to the uneven polypeptide lengths.

Deglycosylation confirmed the presence of the ∼20 kDa carbohydrate mass expected for HCMV gB produced in insect cells (Fig. 4C). Digestion by PNGase F after FL-gB denaturation caused an elecrophoretic shift in all of the three protein bands, releasing the 100 and 50 kDa polypeptide backbones of, respectively, the uncleaved precursor and the SU/TM chains, -with the two chains co-migrating as a single band. Quantitative deglycosylation by PNGase F also showed that recombinant FL-gB was essentially free of core fucosylation. Combinatorial analysis in native conditions by endoglycosidase F isoforms did not detect complex biantennary –Endo F2-sensitive– sugars, whereas TM chain was modified by only Endo F1-sensitive glycans (oligomannose and/or hybrid sugar trees). Full native deglycosylation of the SU chain/moiety required both Endo F1 and F3, showing steric cross-hindrance of the respective glycan substrates within the large sugar shell surrounding this region of HCMV gB. At the same time, micro-heterogeneity in SU N-glycosylation was observed by Endo F1 digestion.

**Figure 5:**
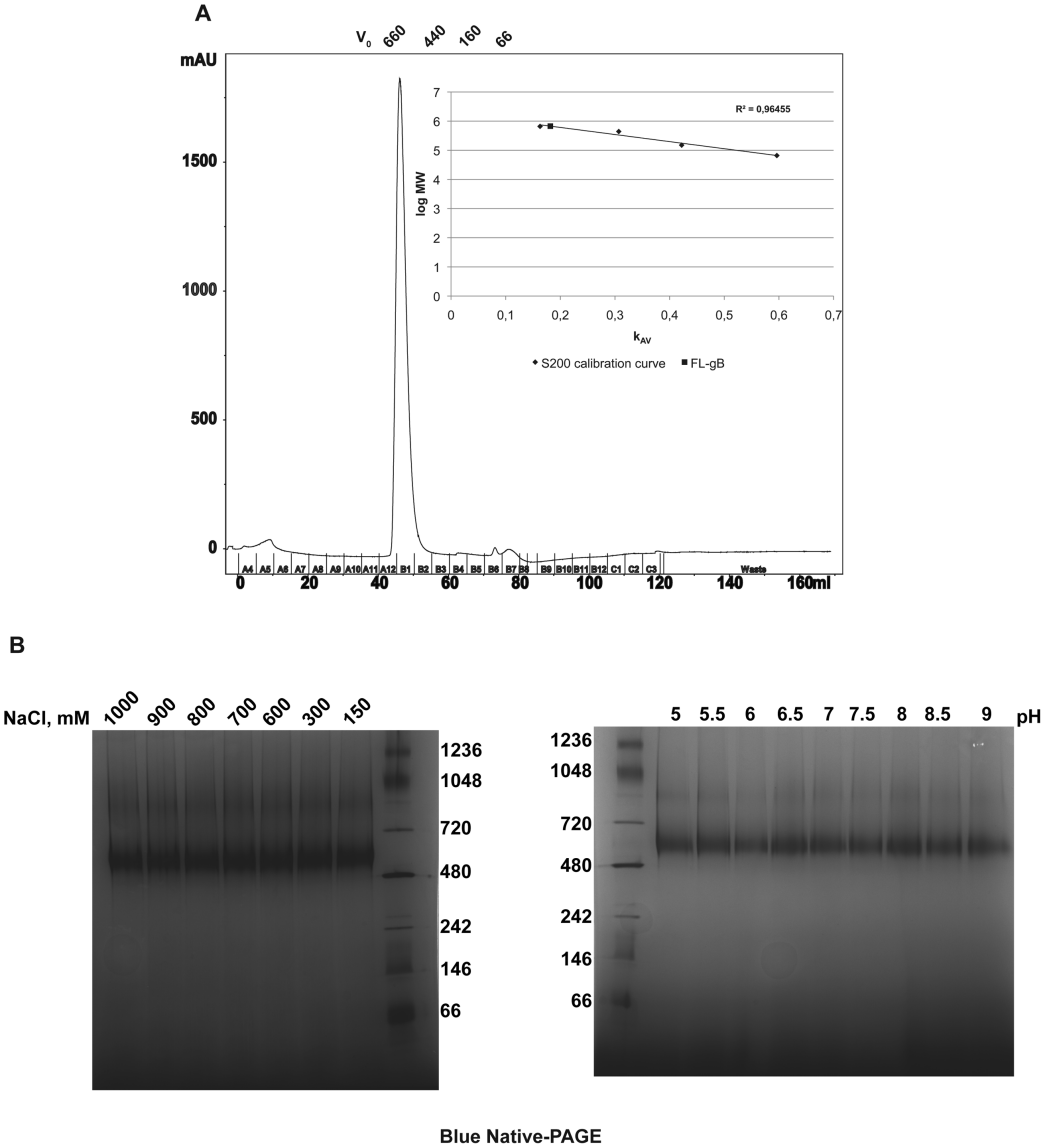
FL-gB is embedded in a large particle. (**A**) Purified FL-gB was injected onto a Superdex 200 pre-packed column equilibrated and calibrated in FL-gB buffer; void volume was measured by Blue Dextran 2000 kDa (V_0_). The retention volumes of the markers are indicated by their molecular weight in kDa. The inset shows the calibration curve and the position of the FL-gB elution peak plotted as K_AV_ against log of the molecular weight. (**B**) 10 µg of FL-gB were incubated in the indicated concentrations of NaCl (left panel) or pH values (right panel) for 1 hour at 37°C, spin centrifuged and the supernatants loaded onto a Blue Native-PAGE gel (native molecular weight markers are indicated as kDa); pH values were verified with a microprobe-equipped pH-meter (Crison).

**Figure 6:**
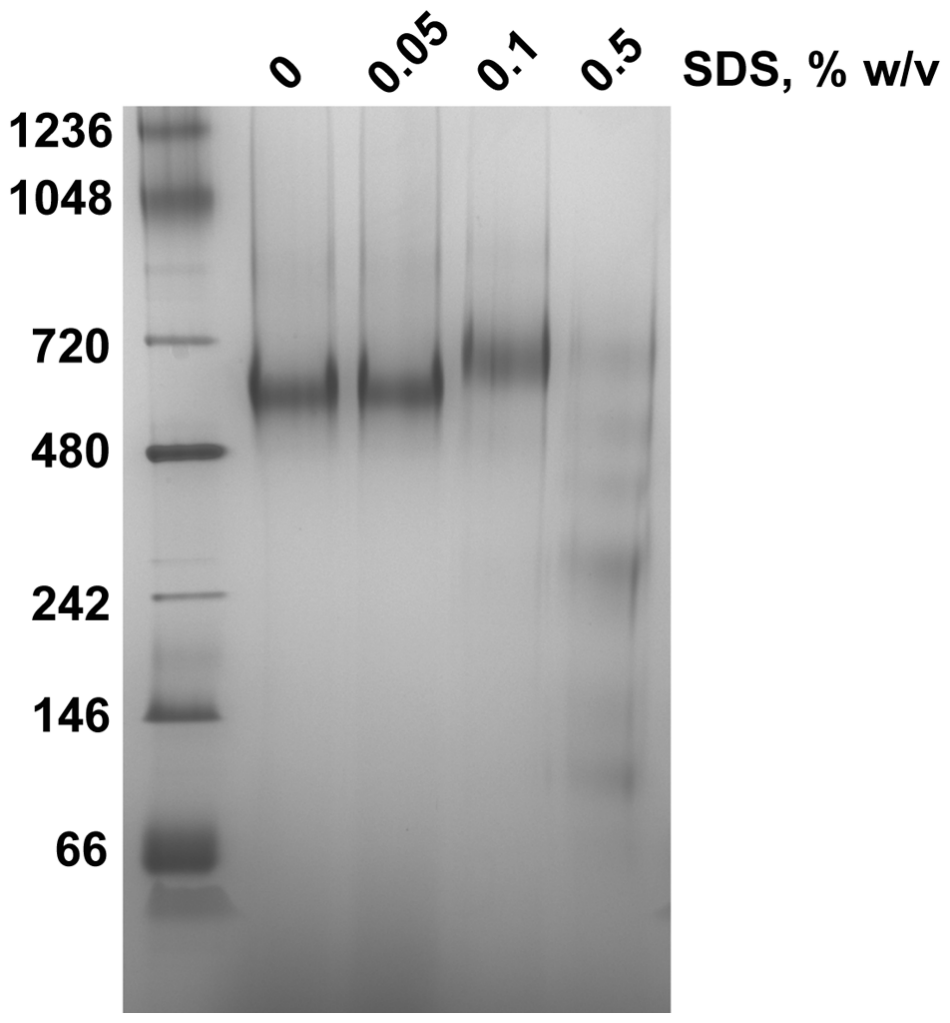
FL-gB particle is sensitive to ionic detergents. 10 µg of FL-gB were incubated for 1 hour at 37°C in the presence of the indicated concentrations of SDS and then loaded onto a Blue Native-PAGE gel.

Efficient sequence specific cleavage and deglycosylation of native FL-gB demonstrated that the protein was obtained in a genuine, non-aggregated state. FL-gB oligomer was characterized as part of a ∼600 kDa particle in solution by size exclusion chromatography (Fig. 5A). FL-gB eluted within the linear separation range in a sharp peak, with a retention volume just larger than the thyroglobulin marker (660 kDa). Size and homogeneity were confirmed at higher resolution and sensitivity by native PAGE (Fig. 5B), where FL-gB migrated as a 590 kDa clear band with a minor amount of a slower migrating form. FL-gB particle was found stable and resistant to high ionic strength and extreme pH values, suggesting that the assembly was insensitive to inter- and intra-molecular gB organization. The molecular weight of cymal-5 micelle (23 kDa) cannot account alone for the mass excess of the particle containing the 360 kDa trimeric gB, which was likely contributed by detergent-resistant host cell lipids. In agreement, a strong dissociating detergent caused the particle to disassemble (Fig. 6). When added at an intermediate concentration, SDS was incorporated into the FL-gB particle whose electrophoretic mobility shifted up in native PAGE. Conversely, the particle dissolved into a ladder of gB-containing micelles when exposed to a higher amount of the anionic detergent.

**Figure 7:**
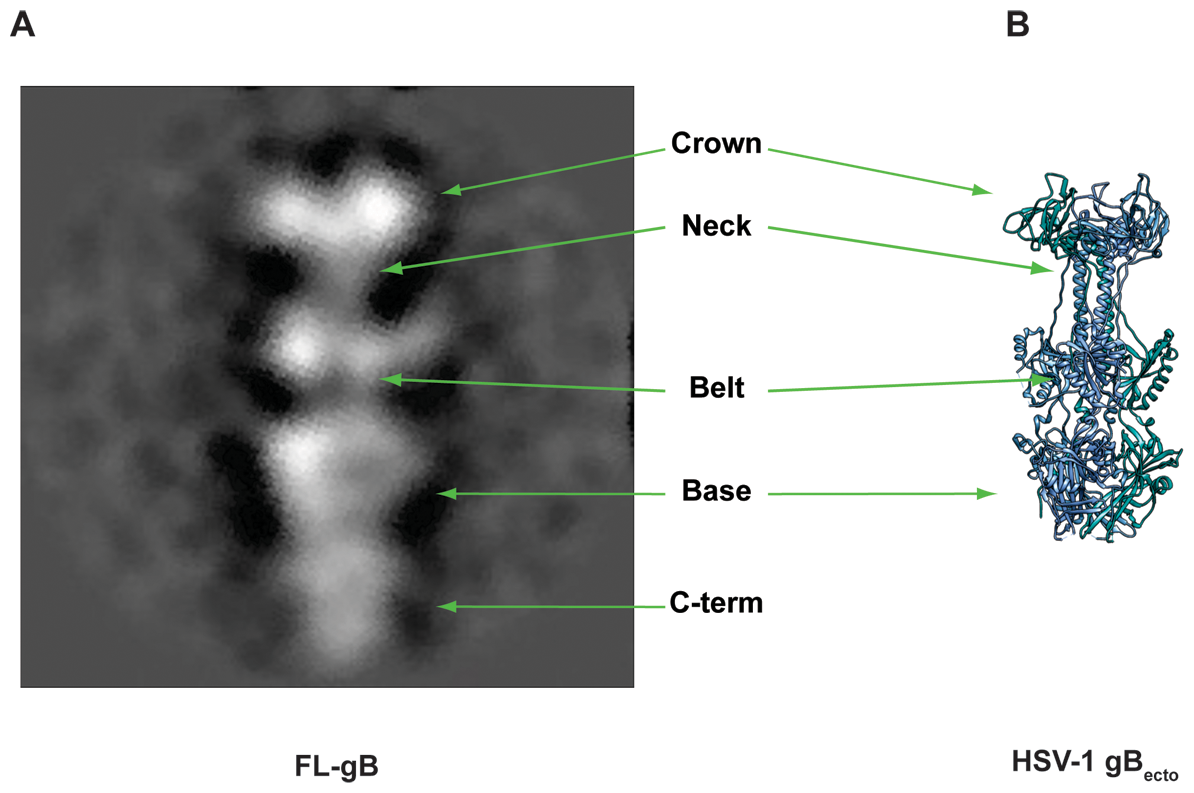
Purified FL-gB is in the post-fusion conformation. (**A**) Negatively-stained FL-gB particles have been imaged by EM and averaged as described in M&M. 2D class obtained is shown. Structural features of gB protein are highlighted. (**B**) Cartoon view of HSV-1 gB_ecto_ trimer (PDB 4HSI) is showed for reference. Dashed lines mark gaps of missing residues in the model.

Electron microscopy (EM) showed FL-gB has been purified in its trimeric post-fusion conformation (Fig. 7). FL-gB ectodomain displays the same fold of gB_ecto_, with the typical apical crown connected to the pre-membrane globular base by an elongated neck and a smaller globular belt, formed by protomer domains IV, I, III and II, respectively [12], [21]. In the structures of HSV-1 and EBV gB_ecto_, the neck clearly arranges into the coiled-coil mentioned above and observed in the BEVS-expressed secreted HCMV gB ectodomain as a heat-labile oligomerization [12], [21], [22]. At the present stage, it is unclear whether its absence in the recombinant FL-gB obtained here reflects any relevant structural difference. Clearly visible in FL-gB is the carboxyl-terminal region comprising the trans- and peri-membrane domains, together packed into a closed globular arrangement and supporting the proposed membrane-dependent folding of gB endodomain [14]. According to their role in the fusion activity, it is tempting to speculate that this arrangement also represents the post-fusion conformation of gB C-term.

Because of the trimeric oligomerization deduced by EM, we infer that purified FL-gB was obtained embedded into a stable bicelle with about 40% of its mass constituted by detergent and host cell lipids.

## Discussion

The easy set-up and robustness of insect cell cultures coupled with the efficient transgene delivery and amplification has made BEVS the preferred choice to pursue a wide variety of difficult-to-express products in a relatively short time frame. However, lytic viral expression systems (VES) provide a basic set of parameters (m.o.i., CCI, TOH) whose intimate cross-influence requires their evaluation as inter-dependent variables.

We undertook a complete factorial screening of the VES core elements in both Sf9 and High Five cell lines for the BEVS-driven expression of full-length mutation-free HCMV glycoprotein B (Fig. 1). FL-gB expression did not display a severe over-expression toxicity or instability since the expression levels were substantially proportional to the viral inocula and testing alternative genetic constructions was then not essential. At the same time, Sf9 cells were not able to provide satisfactory volumetric yields showing a marked inverse relationship between specific expression level and cell concentration. We interpreted this result as the indication that FL-gB biosynthesis demands a high metabolic support. This speculation is based on the evidence that Sf9 cell density effect has a metabolic origin and it can be amended by metabolic manipulation to increase Baculovirus progeny titers [30]. Nevertheless, improved viral growth was not correlated with enhanced transgene expression (Carinhas N., unpublished results), pointing out that protein over-production is somewhat dichotomic to viral replication. On the other hand, High Five cells were able to sustain FL-gB accumulation at a higher degree. Coherent with the often reported better performance in BEVS, a stronger induction of metabolic pathways has been recently observed in High Five compared to Sf9 by *Ac*MNPV infection [31]. The practical consequence of the increased metabolic rates is the quantitative consumption of glucose and glutamine along with a high oxygen uptake by infected High Five. Overlooking this phenotype may ultimately lead to the undesired exhaustion of the culture medium and a consequent poor performance of the system.

The evidence that FL-gB intracellular accumulation arrested early in the infection kinetics prompted the extension of the identified initial conditions into the orthogonal evaluation of candidate additives. Both rapamycin and cysteine increase FL-gB expression over the basal level (Fig. 2 and 3). Their combined use seemed to be additive, although we have applied a relative approach to evaluate gB expression. In this respect, we cannot formally conclude that the two strategies are truly non-synergistic and then acting on related bottlenecks. Nevertheless, a strong synergy is unlikely, since it would be also evident at a qualitative level. The effect of rapamycin was particularly intriguing by showing an infection phase-dependent effect on gB expression, along with the uncoupling of Baculovirus growth and host cell swelling. It is worth to note that the *Ac*MNPV envelope protein gp64 (belonging to the same gB structural class III of viral fusion factors) displayed also an early flat expression in saturating infections. The expression profile of gp64 was insensitive to rapamycin in accordance with the viral growth phenotype, showing that TOR inhibition does not induce a generic increase in Baculovirus-driven expression. This evidence indicates that rapamycin does not represent an expression booster of general use in the BEVS. Thus, gp64 topogenesis might be co-ordinated with the overall timing of the autologous viral cycle and its expression seems not influenced by alterations ineffective on the infection cycle. Indeed, we favour the hypothesis that FL-gB benefits from rapamycin by a seeming paradox effect of saved biosynthetic potential for a demanding recombinant protein upon inhibition of the infection-induced global translation burst. Cell size growth is mediated by TORC1-S6K through the up-regulation of ribosome biogenesis in response to nutrients and growth factors [26], [32]. Hijacking mTOR hub turned out one of the several attractors in the evolution of intracellular pathogens which can manipulate this network by rapamycin-resistant perturbations [33]. We report that Baculoviruses also evolved a similar strategy by showing an infection phase-dependent sensitivity to rapamycin inhibition of virus-induced cell size increase. The broad implications and the underlying mechanism of discovering that cell swelling is a dispensable epi-phenomenon in a viral infection are beyond the scope of the present work. Further investigations, however, will disclose to which extent this observation can be useful to BEVS technology.

Despite the secreted gB ectodomain has been considered an easier target than the full-length form, final yields of both BEVS-driven FL-gB and the fusion loop-deficient gB_ecto_ variant [22] are remarkably close (0.35 FL-gB mg/L against the reported ∼0.5 gB706-4M mg/L). These similar productivities strongly suggest that ectodomain folding represents a bottleneck for gB over-expression. Coherently with this consideration, cysteine supplementation shifted the rapamycin-induced extra FL-gB insoluble amount to the detergent-extracted fraction, confirming that achieving a stable fold is a limiting step in wt gB biosynthesis.

As the result of the above multi-modal approach, we report that a wild type Class III viral fusion factor can be produced recombinant in the milligram range (Fig. 4–6). FL-gB was obtained as a stable 600 kDa particle, considerably larger than the size of the full-length gB trimer. The particle was not an amorphous protein aggregate as by its mono-dispersed appearance and because purified FL-gB was an efficient substrate for two different types of modifying enzymes. Therefore, we speculate that the mass exceeding the 360 kDa FL-gB molecular weight is partly made by a cell-derived detergent-resistant lipid domain gB is stably interacting with. This hypothesis is corroborated by the previously observed gB association with lipid rafts [34] along with the evidence provided here that post-fusion FL-gB particle collapses in the presence of an ionic detergent in non-denaturing conditions. It will be interesting to investigate in the near future gB-lipid interactions at a deeper level, and the role they play in gB activity. Additionally, furin-cleaved and -uncleaved FL-gB were indistinguishable as the two forms migrated together in the native state, confirming previous reports that this post-translational modification has not evident structural roles and is dispensable for HCMV infectivity [22], [35].

Moreover, the evidence is provided that a wild type sequence is not self-sufficient for gB to maintain the pre-fusion state (Fig. 7). Hence, it is plausible that the purified FL-gB obtained here is antigenically equivalent to the gB_ecto_ variants evaluated so far [6], [36], [37].

The careful analysis reported here allowed to find out and overcome the bottlenecks in the expression of recombinant full-length gB. These results will help to identify the determinants required for preserving gB active pre-fusion fold and the structure-function relationships of HCMV virion fusion machinery.
